# Opium and Alcohol

**Published:** 1908-05-09

**Authors:** 


### The Hospital

A Journal of

The Medical Sciences and Hospital Administration.

New Series. No. 60, Vol. III. [No. 1132, Vol. XLIV.] SATURDAY, MAY 9, 1908.

### Opium and Alcohol.

In November 1906 the world received the surprising intelligence that the Chinese Government had approved the regulations recommended by a certain Tong Shao-yi for the gradual suppression of opium cultivation and consumption in the Chinese Empire. The new edict has now been in force for some eighteen months, but little had been heard of the effect of its operation prior to the appearance of the article from the Shanghai correspondent of the *Times* which was published some weeks ago. Now that we ourselves are again in the throes of a struggle with our own analogous problem of alcohol, this experiment at legislative interference with a fixed habit of a people little prone to such departures presents an element of personal interest. At the same time we have to recognise that between the opium problem of the East and the alcohol problem of the West there are certain abiding distinctions. In the first place there is no ground for believing that the use of alcohol can on any score be considered a necessity for the maintenance of health in this country. In the East, on the other hand, there exists a widespread belief that opium is vitally necessary for the inhabitants of malarious districts. Whether this belief be justified or not, there is no doubt of its wide currency, and its existence complicates the problem. In the second place, the abuse of opium is far more limited in its evil effects than is the case with alcohol, for the opium-drunkard does not exhibit his demoralising presence abroad, nor does he incline so much to crimes of violence as does his alcoholic brother.

But if in some respects the abuse of opium is less damaging to the public welfare than the abuse of alcohol, human nature is sufficiently alike in the Orient and Occident respectively to permit a parallel between the problems these drugs present. In each case the prime aim of legislation is to prevent the excessive use of a substance whose temperate employment is harmless if not actually beneficial; in each case, also, new legislation encounters ingrained habits sanctified by hundreds of years of tradition, habits interference with which is likely to be regarded as a personal affront both by the temperate and the intemperate. In the light of these considerations the tentative estimate formed by the *Times* correspondent of the influence to be attributed to the Chinese edict deserves to be taken to heart. This writer emphasises the existence of a "strong and continuing impulse from the people, and especially the educated classes, against the use of opium, and an earnest effort on the part of the officials in many districts to suppress it." At the same time he makes a natural comment upon the optimism of those who "rightly despair of Utopia in their own neighbourhood to look for it on the other side of the world": the benevolent community among us who "are able with little effort to imagine the opium habit of some hundred million Chinese reformed by a stroke of the Vermilion Pencil." He points out that the only acceptable evidence of the sincerity and success of the edict is to be looked for in a reduction of the home-grown supply of opium, and is able to report one district only in which a definite limitation has occurred. Elsewhere the edict is a dead letter, or is literally fulfilled by returning the area under cultivation at some 20 to 30 per cent. above the actual figure, thus allowing an ample margin for paper reductions without interfering with the cultivators. The most hopeful point to be detected is that, in the opinion of respectable Chinese, opium-smoking in public has become not only illegal, but bad form, and that young men in the tea-shops and other places of entertainment are no longer tempted to acquire the habit.

If this estimate be a just one, it corroborates the experience of other countries which have attempted legislation in advance of the public morals of the time. Unfortunately for us the complex interests connected with our liquor traffic have brought into the political net a problem which to the dispassionate eye is purely educational. Although here and there an individual may be coerced into sobriety by restrictive enactments, the growth of moral sense alone can make a people self-controlled in the matter of sensual pleasures. Such an assertion is a truism of moral philosophy, let politicians say what they will. No body of men is more constantly faced with the disastrous effects of alcoholic excess than our own profession, and none, we venture to say, desires temperance more eagerly. And well it is that we should have this clear perception of the evils to be attacked, for we have unsurpassed opportunities of uttering the word in season, and of preaching the gospel of physical efficiency under circumstances favourable to the influencing of men. This being the case, we may be content to leave politicians to quarrel over royal roads to temperance, while we continue to bring home to the people how valuable is health and how sure are the penalties of over-indulgence. Happily we have not to create the "strong and continuing impulse" of which the *Times* correspondent speaks. This is already a flourishing plant among us. Even within the memory of men still young sobriety has made marvellous strides, and it is no over-sanguine belief that the sober individual of to-day forecasts a sober family in the next generation. Altogether the outlook in this respect is decidedly encouraging, for the increase in temperance is the result neither of an emotional outburst nor of legislation, but of a reasoned appreciation of the evils of excess, and rests therefore on the firmest foundations. Under these circumstances, if we may not relax our efforts, we may regard the future with equanimity, whatever be the fate of the liquor legislation now before the country. Moral betterment floating in on an emotional wave like that of the Puritan *régime* is unstable, and, as history tells us, prone to end in disastrous reaction; but a movement based on reason is firmly founded, and we need not fear a set-back even from the most advanced propositions of extremists in temperance.

